# MacGyver Neuroscience: Cost-Effective Laboratory Exercises for Pre-Health Education

**DOI:** 10.59390/001c.161862

**Published:** 2026-05-27

**Authors:** Patrick Foo

**Affiliations:** 1 Psychology & Neuroscience University of North Carolina at Asheville

**Keywords:** neuroscience pedagogy, neuroanatomy, medical education, virtual simulations, introductory courses, neuroscience minor

## Abstract

Developing effective Pre-Health curricula can be a challenge at primarily undergraduate institutions (PUIs). Limited course offerings, tight budgets, heterogeneous student populations, and ambitious learning outcomes pose challenges that will not likely diminish in the near future. Here we present a series of laboratory exercises embedded into a Neuroscience Minor program that are designed to introduce undergraduates to basic surgical skills, safety protocols, mammalian neuroanatomy, and endoscopic techniques. These low-cost laboratory activities can effectively illustrate applications of Neuroscience knowledge in various health care professions.

The Society for Neuroscience (SfN) was founded in 1969, supporting the successful development and growth of our field. Neuroscience is now an established major course of study in hundreds of undergraduate institutions across the nation. More recently, six core competencies have been identified for these programs, including (1) critical and integrative thinking, (2) oral/visual/writing communication skills, (3) articulating the interdisciplinary and interdependent nature of neuroscience, (4) quantitative reasoning, (5) experimental design, and (6) appreciation for how neuroscience can contribute to global solutions (Ramirez, 2020; Wiertelak et al., 2018). Despite funding and support challenges, we have created a successful Neuroscience program at a PUI state university in the southern United States.

The Neuroscience Minor was established at UNCA in 2009 and is a 21 credit-hour program requiring courses in Psychology, Cellular/Molecular Biology, and General Chemistry, with available electives in Health Sciences (formerly Health and Wellness Promotion), Mathematics and other departments. Broadly, our program has two complementary goals - the first focuses on teaching the aforementioned core competencies to undergraduates (see for example, Kaur, 2022), and another that addresses professional training for pre-health students.

At our university, students can major in traditional disciplines or create their own individual degree program as part of their preparation prior to applying to Medical, Dental, Physician’s Assistant, and other Allied Health graduate schools. Approximately a third of our current student population in the minor (~90 students total) self-identify as such. Working closely with UNCA’s Pre-Health Advisory Committee, we aim to add value to their education with a minor that will supplement their course of study. With only two full-time faculty, and in the face of ongoing funding challenges (see Reiness, 2012; Wang et al., 2024), we have “MacGyvered” a cost-effective set of eight laboratory exercises which are presented here.

## DESCRIPTION OF LABORATORY EXERCISES

### Labs 1-3: Fundamentals of Neuroscience

Students in our minor program are required to complete Fundamentals of Neuroscience, a 200-level survey course that was originally developed (and remains cross-listed) as a lower-division elective in the Psychology major. We use the first two parts of, “Neuroscience: Exploring the Brain” as the textbook for this class (Bear et al., 2016). Major topics include the history of the field, neuroanatomy, neuron doctrine, electrophysiology, neuropharmacology, synaptic transmission, perceptual systems, and basic motor functions. We aim to introduce the idea that our very young discipline represents a promising interdisciplinary and interdependent perspective that can provide testable answers to longstanding questions about the human mind. Thus, we aim to develop the third and sixth core competencies listed above for this mixed student population.

To effectively deliver course material, we supplement lectures with accessible active learning exercises (e.g. Meadows et al., 2024; Munoz & Boucher, 2025). Focusing on the remaining core competencies, we previously performed two traditional laboratory exercises that employed SpikerBoxes to observe cockroach action-potentials and human EMG recordings (see Gilbertson et al., 2022; Marzullo & Gage, 2012). These signals were transcribed using the “Spike Recorder” oscilloscope phone app. Students worked in pairs to complete basic data analysis and write a traditional lab report that was evaluated with materials from the Association of American Colleges and Universities (AAC&U written communication and critical thinking value rubrics).

However, obtaining reliable and accurate data from SpikerBoxes remains challenging. Therefore, we have moved to using virtual surgery simulations to bridge basic theoretical concepts with familiar therapeutic applications (see competencies 3 and 6). After sampling several phone apps, such as Dissection Master XR and Visible Body, we chose Touch Surgery by Medtronic. This free program simulates a wide range of surgical techniques from Dentistry, Orthopedics, to Neurosurgical procedures.

We employ three of these simulations at strategic points in the semester (e.g. after a midterm exam) to buoy student enthusiasm and teach medical procedures and terminology (Arantes et al., 2018). Each simulation is accompanied by a learning comprehension worksheet, with short-answer questions addressing indications for the procedure, potential risks/benefits, step-by-step techniques, and typical patient outcomes (Meir, 2022). Additionally, an open-ended essay prompt, “what was the most important thing you learned in this lab?” is always included. These activities allow us to introduce medical topics including maintaining hemostasis, minimizing surgical infections, and the importance of following surgical procedures in an introductory class. The labs are described briefly here, and more information is contained in the Appendix.

#### Lab 1: Lumbar Puncture simulation

The first simulation assignment covers Lumbar puncture, which many students are exposed to in media portrayals like “Grey’s Anatomy” or “The Pitt.” The app teaches the procedure in multiple steps and lists the four common central nervous system disorders that can be diagnosed by the lumbar puncture (Meningitis, Encephalitis, subarachnoid hemorrhage SAH, and idiopathic intracranial hypertension). Worksheet questions address the latter, and ask technical questions about the former including, “Where do you place your thumbs to locate the puncture site?” (bilaterally at the patient’s L3 and L4 vertebrae) and “Why does one angle the 22-gauge needle when inserting into the spine?” (to separate dural fibers instead of cutting them).

#### Learning outcomes

Describe how Germ Theory translates into antiseptic procedures, identify components of the human Cerebral Spinal Fluid system and the value of sampling CSF for differential diagnosis of diseases.

#### Lab 2: Cataract Surgery simulation

The second exercise covers a procedure that many students’ relatives have experienced: Lens replacement surgery for cataracts. Students are often surprised that diabetes, steroid use, dehydration, UV light exposure, poor nutrition, smoking, alcohol use, and low SES are contributing lifestyle factors to developing cataracts. This lab is assigned when our lectures cover visual system anatomy and procedures involved in a typical optometrist visit. In addition to similar procedural questions in the lumbar puncture lab, here students learn about secondary uses for surgical tools in the service of patient safety (e.g. placing a nucleus chopper posterior to the phacoemulsification probe to protect the posterior capsule of the eye).

#### Learning outcomes

Identify the main anatomical components of the human eye. Describe lifestyle factors that contribute to good visual health, compare cataract surgery to LASIK and radial keratotomy of the cornea.

#### Lab 3: Acute Craniotomy simulation

Finally, students learn about Acute Craniotomy surgery which is employed after severe TBI (Traumatic Brain Injury), aneurysmal SAH, and malignant edema following a stroke. This prolonged surgery involves decompressing the brain by safely removing a section of skull. Students often comment in their lab reports about the overlap between this modern version of trepanation and practical skills used in applied industrial/culinary arts like carpentry and cooking (e.g. using Raney clips to control scalp bleeding, having to cool drill bits while perforating the skull, or using wax to prevent exposed bones from dehydrating).

#### Learning outcomes

Identify the meninges of the Central Nervous System and the anatomy of the human skull. Compare treatments for mild and severe TBI and consider youth athletics concussion protocols across different sports.

### Labs 4-8: Advanced Neuroscience

Advanced Neuroscience is offered as an upper-division elective in both the Neuroscience minor and Psychology major and is designed for students pursuing careers in the Pre-Health disciplines. This course was previously offered once every academic year; recently we’ve doubled this frequency as demand has increased. The content of this course extends what is covered in the prerequisite Fundamentals course, first reviewing and extending neuroanatomical knowledge (always a foundation of medical knowledge, see Arantes et al., 2018; Casimo et al., 2022; Wang et al., 2024) via the appendix of Ch7 and then covering functions of the brain including emotion, learning, attention, and memory in parts 3 and 4 of the Bear et al., text. Furthermore, in this true laboratory class, we replicate and extend the previous Fundamentals of Neuroscience SpikerBox labs with five new topics. With these low-cost exercises, we aim to train critical thinking, experimental design, communication, and other “durable skills.” Students write traditional lab reports for each of these exercises, which are evaluated with the aforementioned AAC&U rubrics. Since the risk associated with these activities is not more than ‘minimal,’ they are considered educational in nature and not subject to IRB review.

#### Lab 4: Cutting and Suturing

Basic surgical techniques like cutting and suturing are fundamental skills in any medical education curricula, and there is a demonstrated benefit to introducing them early in one’s education (i.e. in the third week of the semester, see Manning et al., 2018). We take this as the opportunity to review safety procedures, including proper use of gloves, eye protection, respiratory masks, and other PPE. Students learn how to attach and remove scalpel blades, along with safe disposal. They also use different scissor and scalpel blade shapes (straight vs. curved) on a number of fruits (grapes, bananas, mandarin oranges, etc.) that simulate various human tissues. Finally, using a mix of in-person and video materials, students are taught how to make simple interrupted and mattress sutures using hemostatic forceps as needle drivers. After practicing on fruits, they do the same on grocery store chicken breasts and thighs as proxies for cadavers (see Braasch et al., 2022; Manning et al., 2018). Material costs include non-sterile sutures and scalpel blades ($5 and 50 cents each, respectively), and market priced produce and meats.

Their final challenge of the lab is to sew a water-tight connection between two lengths of rubber inner tubes (we use large-diameter mountain bicycle tires). This simulates the challenge of repairing damaged blood vessels, which remained an unsolved problem until the end of the 19^th^ century. Then, even best practices like using tubes of metal or bone to surround the damaged artery often led to infection, leakage, and gangrene.

#### Learning outcomes

Introduce safety procedures, various cutting tools and techniques, and simple interrupted and mattress sutures. Student lab reports are evaluated using the AAC&U rubrics, giving feedback on their critical thinking and science communication skills. To date, no student has mastered the inner tube challenge, and we reserve the solution as a cliffhanger until the reports are returned during lecture the following week. This affords the opportunity to discuss how Dr. Carrel adapted his mother’s technique of sewing blouse sleeves to win the Nobel Prize in 1912.

#### Lab 5: Cow Eye Dissection

A cow eye is very similar to the eye of a human, but the techniques required to dissect it are relatively simple and safe, so we use this as an introduction to dissecting preserved tissue from a scientific supply company ($5 per specimen). Since students in this class have already performed a “Touch Surgery” simulation in the prerequisite Fundamentals class, this allows us to extend their previous knowledge to discuss laser eye surgeries (PRK & LASIK correction), cataracts, glaucoma, retinal detachment, and the relatively poor night vision of humans. It is also the first lab that students are confronted with tissue, the smell of preservatives, etc.

Our crude scalpels and scissors have nowhere near the sharpness of a surgical keratome but can be illustrative of the differential designs of slicing, piercing, and cutting tools. When removing the lens of the eye, students can be disappointed to find it an inflexible, hard marble vs. what is described in their textbook as a flexible liquid-filled sphere, and this allows for discussion of preservation techniques in their lab reports. Importantly, it also gives the instructor a good opportunity to assess whether the PPE, safety, and techniques from the previous lab have been ingrained or if further review is necessary.

#### Learning outcomes

Learn mammalian eye anatomy in a preserved specimen, compare living structures to textbook examples, understand the pros/cons of different preservation techniques, compare and contrast this lab to virtual surgery simulations.

#### Lab 6: Civil War Brain Dissection

Originally, this lab consisted of straightforward dissection of a preserved sheep brain, performed in pairs to section the brain along sagittal, coronal, and horizontal sections. Students would identify and label the spinal cord, hindbrain (medulla, cerebellum, pons, fourth ventricle), midbrain (superior and inferior colliculi of tectum), diencephalon (thalamus, hypothalamus, pituitary, pineal gland), and telencephalon (basal ganglia, limbic system, corpus callosum, and the four lobes of the neocortex). However, we’ve become dissatisfied with this approach as it was too easy to complete and have developed what we think is a more creative and active learning approach.

We call this, “Civil War” brain surgery, and use it to discuss in lecture the history of our field, and to illustrate the great difficulties faced by Neurosurgeons until the very recent past. Instead of separated sheep brains for each pair of students, we purchase preserved brains in their skulls with dura intact ($20 each). Furthermore, we only allow tools that existed in the late nineteenth century (e.g. manual bone saws or rongeurs) and prohibit use of photography for lab reports. Instead, one must use their innate artistic and observational skills to describe their findings (similar to Casimo et al., 2022). Students are encouraged to try preserving functional units (e.g. dissecting the intact hippocampus) vs. performing cross-sectional coronal and sagittal slices of the brain.

#### Learning outcomes

**Figure 1.**
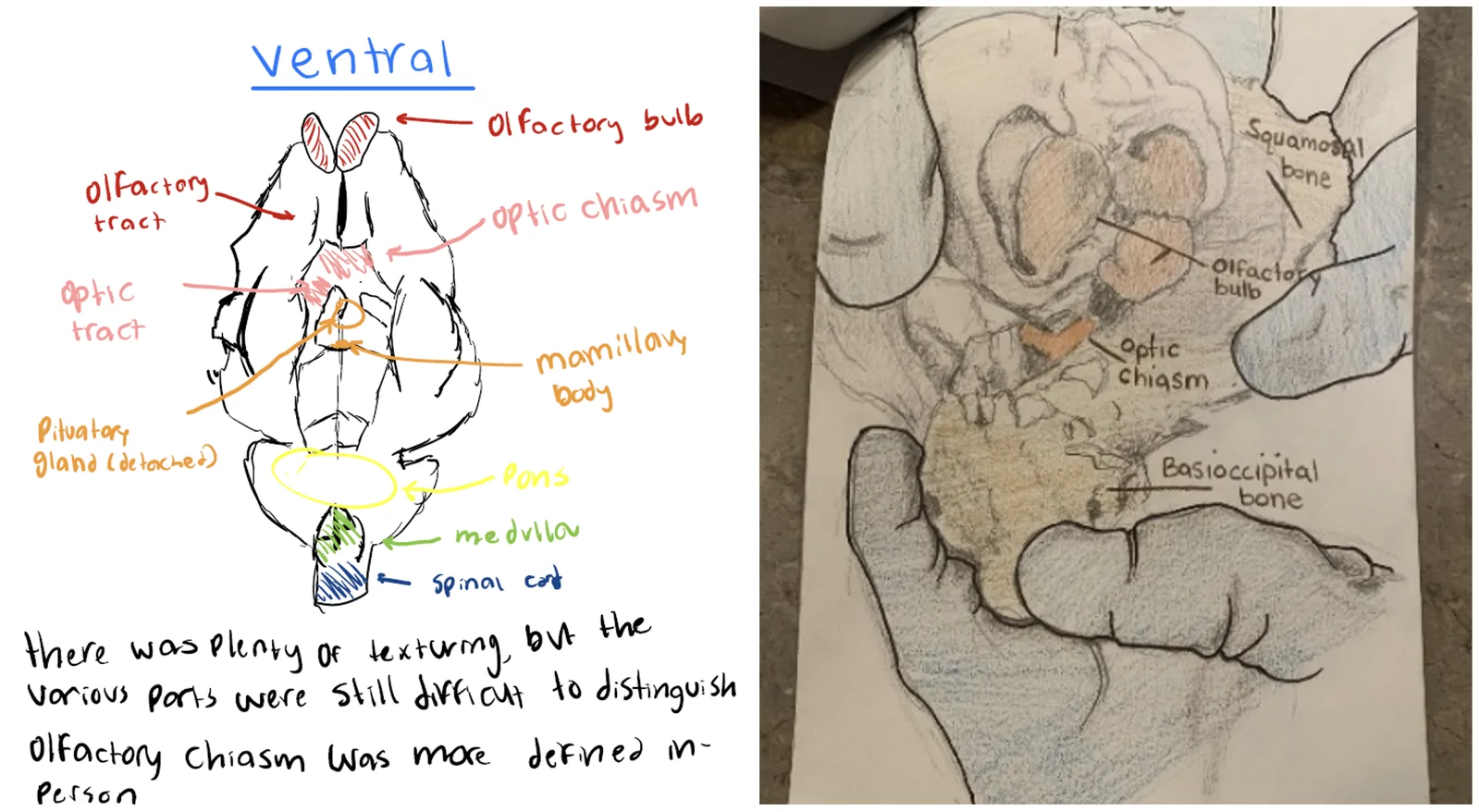
Two examples of Civil War brain dissection illustrations

Learn how to access the brain through the skull, recognizing the difficulty in successfully accessing the brain without damaging it. Practice effective cooperative work (e.g. stabilizing the skull for their partner who is cutting), and how to do it safely. Observe the difference in skull morphology between ventral vs. dorsal aspects, attempt to draw and describe their methods and results (Figure 1).

#### Lab 7: Hog Head dissection

Since Asheville, NC is nationally known for its long-standing farm-to-table gourmet tradition we are able to source locally-produced meats which we use as laboratory specimens. We purchase pig/hog heads from a local butcher shop within hours of harvest ($30 each). Thus, we can extend the previous labs with a “fresh brain” for dissection and exploration. This has the obvious advantage that structures like the lens of the eye and the cortex retain their natural texture and consistency; additionally, since these are literally food-grade products, we can reduce exposure to biological preservatives.

We preface this particular lab with a discussion on ethical harvesting and experimentation practices. We discuss the antiseptic merits of surgical draping, and about potential psychological benefits draping can afford when working on “live” tissue. Additionally, we employ a very student centered active learning approach by this point in the course, giving students the responsibility for selecting, organizing, and integrating their prior knowledge into a unique surgical plan of action (Meadows et al., 2024; Ramirez, 2020). For example, pre-dental students can practice tooth extraction, explore mouth and throat anatomy, and cranial nerve organization. Students interested in Otorhinolaryngology may focus on nasal cavity, sinus, or middle ear anatomy. Potential Optometrists often revisit the eye and its structures, and all can benefit from additional suturing practice on actual cadaveric tissue (Braasch et al., 2022). The instructor functions as a “guide-on-the-side” during this capstone experience (Ramirez, 2020).

Since this lab is performed during lectures covering the history of mental illness, we encourage interested students to try their hand at psychosurgery (see Lichterman et al., 2022). In addition to the hand tools from the Civil War lab, we introduce rotary drills so they can compare leucotomy and lobotomy techniques.

#### Learning outcomes

Introduce discussions of ethics, professional empathy, and bedside manner. Compare near-live tissue with preserved specimens, contrast virtual simulations with hands-on techniques. Evaluate the effectiveness of power vs. hand tools for precision and accuracy, learn specialized pre-health anatomy, compare Freeman’s and Moniz’s approaches to psychosurgery in the 20th century.

#### Lab 8: Endoscope

Recent advances in medicine and surgery include interventional flexible endoscopic alternatives for visualization and surgical manipulation (e.g. colonoscopy, gynoscopy, and arthroscopy; see Shim et al., 2017). However, this equipment remains expensive and hard to obtain for primarily undergraduate institutions like ours. While virtual simulations like TouchSurgery, Endoscopy3D and GastroEX offer students lifelike visualizations, they do not expose students to the more important hand-eye coordination and partner interactions inherent in many of these techniques (Foo & Ruiz, 2019; Moreira-Pinto et al., 2013).

**Figure 2.**
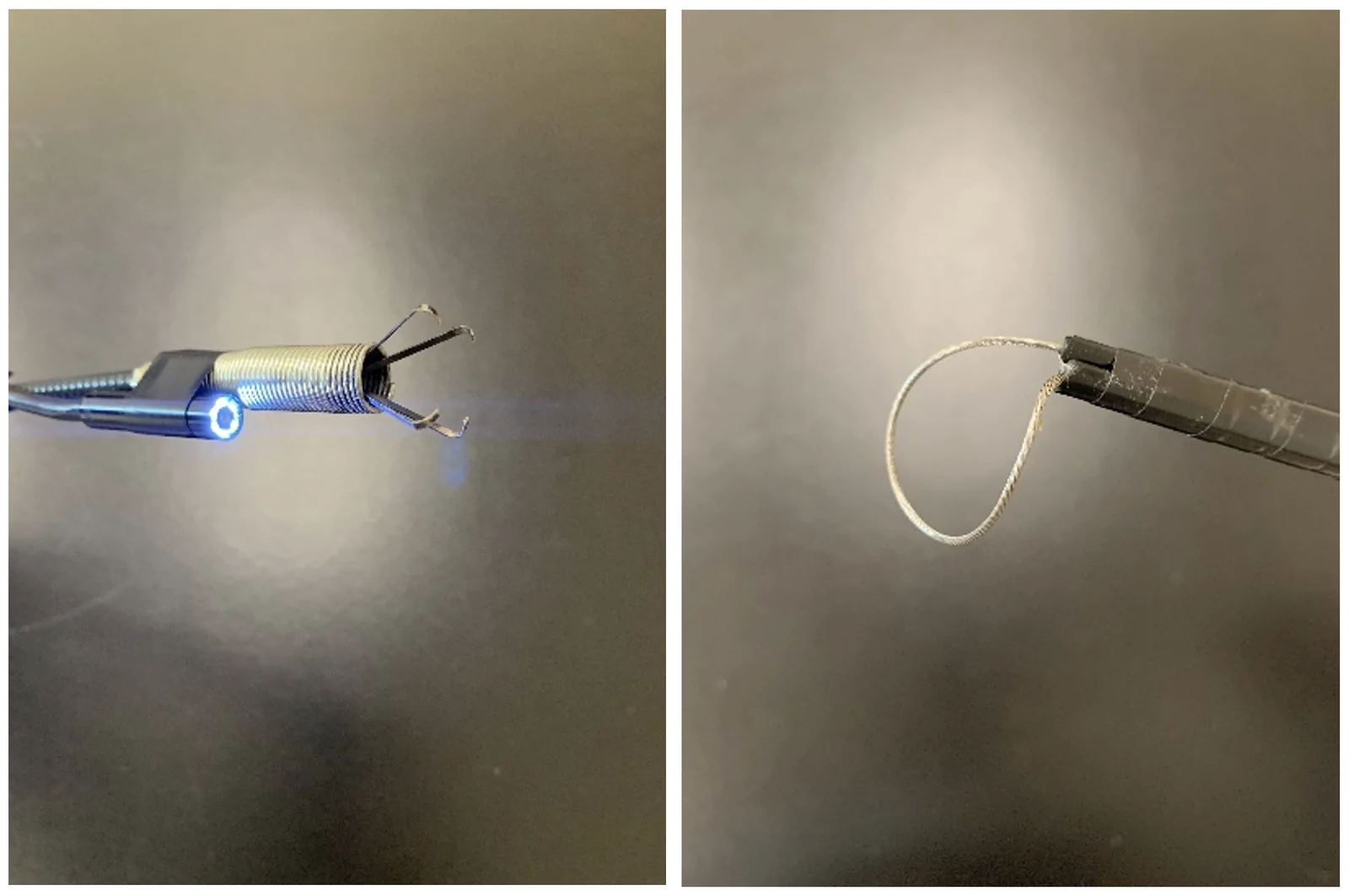
Endoscope camera/grasper, and surgical snare used in lab #8.

Instead, we have combined an inexpensive, $32 BlueFire semi-rigid flexible wireless inspection camera with a $15 plumbing grasper (simulating a Babcock forcep/clamp) to introduce students to the basics of endoscopic visualization and navigation. Extending these tools, we have recently developed a snare built from basic bicycle parts (e.g. derailleur cable housing, brake cables, and wooden dowels – approximately $5 per finished unit) that simulates a scaled-up Endoloop ligature (Figure 2).

Working in pairs, students begin with a simple scavenger hunt for items in a closed cardboard box (e.g. jellybeans, plastic toys, etc.) and work their way up to finding and removing items (e.g. a specific grape in a cluster, the head of a gummi bear, etc.) that simulate tumorous tissue. Since only one partner controls the visual input but both must cooperate to stabilize and manipulate the target, these activities require close/effective collaboration, communication, and coordination skills for successful completion.

#### Learning outcomes

Simulate modern surgical techniques. Gain practice in the perceptual-action coordination required for remote surgery. Discuss (dis)advantages of patient outcomes, and practice cooperative coordination between partners.

## RESULTS

### Quantitative and Qualitative Feedback from Students: Fundamentals of Neuroscience

Students provided feedback about the labs using a Likert-type scale (see Tables 1 and 2) from a university-wide evaluation form at the end of the term. The specific prompt was, “My instructor provided assignments and/or activities that enabled me to better understand the principles introduced in class.” The first table reports from 151 Fundamentals of Neuroscience students from Fall 2020-present with a 49% response rate (74 students). Note that a different form was used in Fall 2024 due to Hurricane Helene and is not reported here.

**Table 1.**
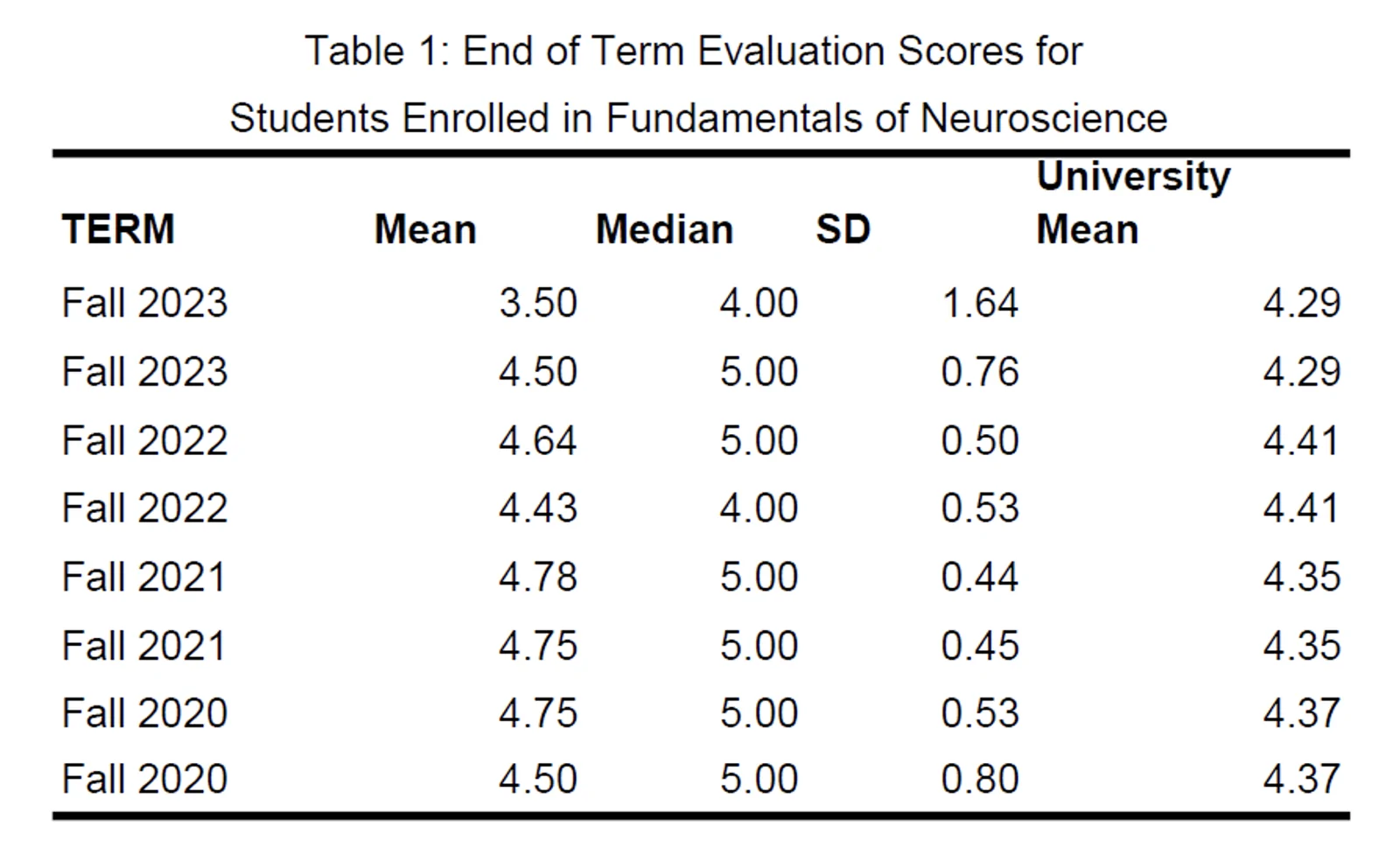
End of Term Evaluation Scores for Students Enrolled in Fundamentals of Neuroscience Note. Scale used was based on 1 (strongly disagree), 2 (disagree), 3 (neutral), 4 (agree), 5 (strongly agree).

With the exception of Fall 2023, where only six students (40% of the class) answered, the above average ratings and spontaneous comments indicated overwhelmingly positive student responses (see Table 1). Additionally, students could write in answers to the open-ended question, “As part of your learning, your class completed several surgery simulations (craniotomy, cataract surgery, lumbar puncture, etc.). How effective were these labs in learning real-world skills and terminology that may help in your future in the health care professions?” Sixty students provided answers to this query which were analyzed to distill common themes (see Leung et al., 2022). Note that an individual student’s response could speak to more than one theme. UNC Asheville Institutional Review Board (IRB) determined the use of paraphrased quotes below as “exempt.”

Many students (13/60 responses) remarked on how the simulation labs highlighted the connection between theoretical concepts and medical procedures:

I valued the opportunity to see how the concepts from lectures and the textbook could be applied in more practical ways. Having a basic understanding of these medical procedures is beneficial, even for students who do not intend to pursue a career as a health care provider.

Moreover, and with the same raw frequency, while some students remarked that they were not planning on a Pre-Health career, others found the simulations a nice introduction to the field:

These labs were very effective because experiences like this are typically not available until graduate or medical school. It was fascinating to observe the detailed complexity involved in these surgical procedures.

Students enjoyed learning surgical and medical vocabulary within the simulation app in the most frequent theme (16/60 responses) observed:

I found the labs very engaging. Even though they were challenging and involved a lot of complex terminology, they did an excellent job of visually demonstrating the concepts. As a visual learner, I especially appreciated having a clear, step-by-step process to follow.

Including these labs at appropriate times in the semester (10/60 responses) appeared to improve student engagement:

The virtual labs helped me better appreciate the complexity involved in medical procedures, and I found them exciting, useful, and worthwhile. Their timing throughout the course felt appropriate and provided an effective way to break up lectures while still reinforcing learning.

Finally, eight of the written responses consisted of positive student reactions to the Touch Surgery app, and many noted that they would continue its use after course completion:

These labs were one of the highlights of the course for me. I hadn’t realized that such advanced educational resources were available online for free. I was even able to use them to show my grandmother what to expect during her upcoming cataract surgery!

### Quantitative and Qualitative Feedback from Students: Advanced Neuroscience

Data from the previous five academic years for the Advanced Neuroscience course is reported in Table 2, consisting of 93 students and a 52% response rate (48 students). Here, aside from two terms (Spring 2023 and 2024) where course ratings were below university means, but well within the SD range, a similar trend of positive student responses is observed.

**Table 2.**
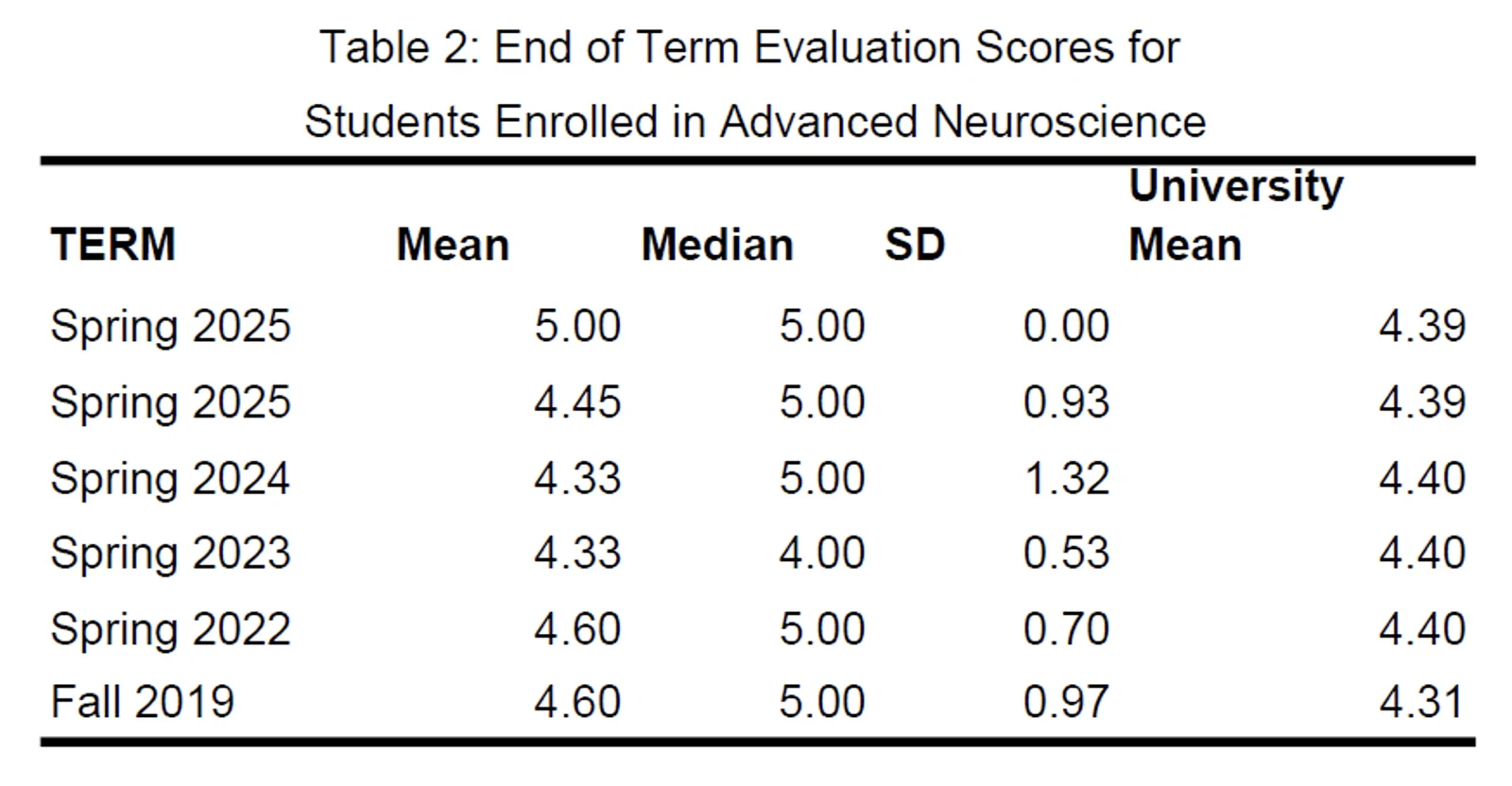
End of Term Evaluation Scores for Students Enrolled in Advanced Neuroscience Note. Scale used was based on 1 (strongly disagree), 2 (disagree), 3 (neutral), 4 (agree), 5 (strongly agree).

Additionally, students could respond to the open-ended question, “Did the laboratory exercises (suturing, cow eye, sheep brain, endoscope, and/or hog head dissections) contribute to your understanding of modern Neuroscience? If so, how?” Below we share themes from 44 student evaluation forms.

Much like their colleagues in the Fundamentals class, students wrote about learning medical terminology (2/44), the simulation app (1/44), student engagement (5/44), Pre-Health careers (4/44), and the contrast between practice and theory (8/44) as below:

These labs were my favorite part of the course because they provided an opportunity to apply theoretical concepts in a hands-on way. While I already found the topics interesting, dissecting a sheep brain and a cow eye made the material feel much more tangible. It was a remarkable experience to actually observe structures like the thalamus and corpus callosum firsthand. The lab reports also helped reinforce the technical terminology and key concepts we learned in class.

Additionally, a few of these eight comments directly addressed the “Civil War” brain dissection:

I feel that I now have a much clearer understanding of the tools and challenges faced by early neuroscientists, particularly through the hog head dissection. The process of opening the skull and working with fresh, fragile brain tissue helped me better grasp the nature of what we are studying.

The increasing difficulty of the semester’s lab activities was not lost upon two observant students:

I observed a significant progression in how my classmates adapted over time. The final lab was especially impressive, and through conversations at our table, it seemed to strengthen some students’ commitment to pursuing paths like medical school, while also boosting others’ confidence in their own abilities.

Many students (6/44 responses) noted the differences between preserved tissues and the fresh specimens provided in the pig/hog lab:

The fresh dissections were eye-opening for me, because I had only experienced preserved specimens previously. Now I know that preservatives substantially change the consistency of tissues, leading to a skewed sense of what to expect in a living patient.

The most common theme (11/44 responses) observed was an appreciation for the student centered pedagogy of the final dissection:

Although it might seem counterintuitive, the more structured labs I’ve experienced in other science courses were often more confusing and left me uncertain about the next steps than the labs in this class. I appreciated the freedom to explore. Being told exactly where and how to cut would have been less engaging and probably less effective. By experimenting, making mistakes, and figuring things out ourselves, we were able to understand why certain approaches worked better than others.

Three comments praised the endoscope lab, and offered suggestions for improvement:

The endoscope lab could be improved by using shorter scopes and better materials like whole watermelons, but it was a nice introduction to modern surgical techniques.

One non-traditional student, a former Green Beret medic praised complimented our explicit treatment of professional standards (1 response):

The explicit modeling and discussion of patient autonomy and dignity helped set the stage for huge gains in both learning (or solidifying previous information) and building physical skills that will translate to better patient care. Not that every student will become a brain surgeon but the increasing confidence with hemostats, scalpels, scissors, and (almost) live tissue will translate very broadly to most fields.

Finally, we were pleasantly surprised to read this one philosophical comment:

I would like to emphasize that working with the sheep brains and conducting the fresh hog head dissections really highlighted how delicate the brain is. These experiences also gave me a deeper appreciation for just how fragile life can be.

## DISCUSSION

In 2009 we started a successful interdisciplinary Neuroscience Minor at an undergraduate university despite relatively modest funding and staffing resources. The goals of this program fall under two broad categories – the first teaches six competencies central to an undergraduate education in Neuroscience (i.e. Ramirez, 2020), and the second supports pre-professional training as outlined by the Association of American Medical Colleges (AAMC) and the American Association of Colleges of Nursing (AACN). We have developed a laboratory curriculum of eight exercises, along with off-the-shelf DIY equipment to support these goals, which should be within reach of the budget of many institutions.

Being able to quickly ramp up simulations from the most basic, like lumbar puncture to acute craniotomy provides our Fundamentals of Neuroscience students a user-friendly overview of theoretical applications and anatomical terminology. Undergraduate students employ their critical thinking, analytical observation, and other “durable skills” to these surgical procedures. This has obvious positive consequences for those proceeding to health careers. Furthermore, retaining the free Touch Surgery app can be beneficial to a broad range of undergraduates. One former student wrote, “shortly after our last VR lab, my father was involved with a work-related accident which resulted in a serious concussion. I was able to explain some of the diagnostic criteria and surgical procedures to my family which made me feel valuable and helped them during a time of real crisis.”

In the Advanced Neuroscience class, we use five stair-stepped lab exercises to introduce modern surgical procedures that span several Pre-Health careers. For some, it deepens their curiosity and confidence as they improve their hand/eye coordination. Former medical students have commented that their suturing and cutting skills were better than their peers upon their first-year rotations. Moreover, while preserved specimens are preferable to textbook images, virtual simulations, and plastic models, they still pale in comparison to the real thing. Confronting the sights, sounds, and smells of surgery can be jarring to students. The comments, “I was reminded of going to the dentist with the bone dust and vibration” and “I really appreciated using Vicks VapoRub to lessen the odor of blood” illustrates these high-impact experiences.

The use of fresh hog and pig specimens provide a natural opportunity to introduce ethical harvesting and animal testing practices. The capstone hog lab can be a venue for future providers to reflect on their own capacities for empathy, compassion, and professional patient care. Some individuals discover that their dream of becoming a “brain surgeon” is not as savory as they imagined, and begin to explore other specialties, or even different careers. Because they can better make these decisions prior to investing in years of professional training we also consider this a potential benefit.

Additionally, in an ongoing undergraduate research project, we are turning our endoscope lab into an interactive exhibit at our local science museum. Children and families have participated in a “Nose Picking Endoscope” contest, complete with a paper mâché nasal cavity, and a “Folding with Forceps” origami challenge. We hope to use these successes to secure funding to improve the endoscope lab with surplus surgical equipment in the future (see Moreira-Pinto et al., 2013).

Performing “Civil War” dissections can better contextualize the history of our field. As most, if not all students are unable to access the brain without destroying it, they come away more sympathetic to the difficulties involved in neurosurgery. Having to observe and draw one’s results like da Vinci or Ramon Cajal dovetails nicely with the overall “renaissance education” mission of our university.

Finally, having the opportunity to perform psychosurgical techniques like leucotomy is often a powerful experience for students. The ease with which an icepick lobotomy is performed is not forgotten quickly and can be a visceral reminder of one of the darkest chapters in our field’s history.

Thus, even with limited resources, by having a “MacGyver” mindset, and focusing on student learning outcomes, one can still provide effective, high impact teaching practices in our field of Neuroscience.

### Address correspondence to:

Dr. Patrick Foo, Psychology Department, 1 University Heights, University of North Carolina Asheville, Asheville, NC, 28804. Email: pfoo@unca.edu

## Supplementary Material

Appendix
