# Supplementary material for: MacGyver Neuroscience: Cost-Effective Laboratory Exercises for Pre-Health Education
Source: J Undergrad Neurosci Educ. 2026 May 27;24(2):25–33. doi: 10.59390/001c.161862 (PMC13625902; doi:10.59390/001c.161862)
Supplement: Supplementary file 1 [file junejournal_2026_24_2_161862_343425.docx]

**APPENDIX 1**

**Additional protocol information about lab exercises.**

The virtual lab exercises (labs 1-3 here) follow step-by-step procedures provided in the Touch Surgery modules. Students use simplified touch screen mechanics to manipulate simulated surgical tools, as on-screen messages supply instructions for each procedure, broken down into phases and steps. For example, lumbar puncture consists of 1 phase and 43 steps, cataract has 4 phases and 95 steps, and acute craniotomy is divided into 3 phases with 150 total steps.

Advanced Neuroscience protocols (labs 4-8) follow well-established curricula used in undergraduate biological sciences and initial medical school instruction. For example, in-person cutting and suturing instruction is augmented by short videos made by the author and available to students via their online learning management system (Braasch et al., 2022; Manning et al., 2018). Similar videos are freely available on YouTube.

The cow eye lab follows the initial steps of virtual cataract surgery (lab 2) and is supplemented by dissection instructions similar to those available from public science museums like the San Francisco Exploratorium. Likewise, instructions for the sheep brain lab (no. 6) can be found on the Carolina Biological Supply website. Since the specimens we use include an intact skull (albeit with an initial cut to the dura level), students in this lab use handheld bone saws and carpenter’s brace drills to access the brain. As such, these procedures have similar risks to activities included in high school woodworking courses.

The hog head lab matches initial steps of the virtual craniotomy simulation (lab 3). Students can replicate suturing or cataract procedures to explore preservative-free qualities of these specimens. Furthermore, they can replicate Moniz’s leucotomy with electric drills and Dremel-style rotary tools. Some students elect to attempt Freeman’s transorbital lobotomy; we use galvanized carpentry nails for their durability and cost. Thus, students assume a similar risk as noted above. Finally, endoscope procedures in lab 8 have been described previously (Foo & Ruiz, 2019).

**REFERENCES**

Braasch, M. C., Minchew, H. M., Riffel, J. D. M., & Berbel, G. (2022). Suture education with soft-embalmed cadavers: A cut above the rest. Kansas Journal of Medicine, 15, 78.

Foo, P., & Ruiz, M. (2019). Inexpensive endoscope activities. Physics Education, 54(5), 055005.

Manning, E. P., Mishall, P. L., Weidmann, M. D., Flax, H., Lan, S., Erlich, M., Burton, W. B., Olson, T. R., & Downie, S. A. (2018). Early and prolonged opportunities to practice suturing increases medical student comfort with suturing during clerkships: Suturing during cadaver dissection. Anatomical Sciences Education, 11(6), 605–612.
